# Exploring and dissecting genome-wide gene expression responses of *Penicillium chrysogenum *to phenylacetic acid consumption and penicillinG production

**DOI:** 10.1186/1471-2164-10-75

**Published:** 2009-02-10

**Authors:** Diana M Harris, Zita A van der Krogt, Paul Klaassen, Leonie M Raamsdonk, Susanne Hage, Marco A van den Berg, Roel AL Bovenberg, Jack T Pronk, Jean-Marc Daran

**Affiliations:** 1 Department of Biotechnology, Delft University of Technology, Julianalaan 67, 2628 BC Delft, The Netherlands; 2 Kluyver Centre for Genomics of Industrial Fermentation, Julianalaan 67, 2628 BC Delft, The Netherlands; 3 DSM Anti-Infectives, DAI/INNO (624-0270), Postbus 425, 2600 AK, Delft, The Netherlands

## Abstract

**Background:**

Since the discovery of the antibacterial activity of penicillin by Fleming 80 years ago, improvements of penicillin titer were essentially achieved by classical strain improvement through mutagenesis and screening. The recent sequencing of *Penicillium chrysogenum *strain Wisconsin1255-54 and the availability of genomics tools such as DNA-microarray offer new perspective.

**Results:**

In studies on β-lactam production by *P. chrysogenum*, addition and omission of a side-chain precursor is commonly used to generate producing and non-producing scenarios. To dissect effects of penicillinG production and of its side-chain precursor phenylacetic acid (PAA), a derivative of a penicillinG high-producing strain without a functional penicillin-biosynthesis gene cluster was constructed. In glucose-limited chemostat cultures of the high-producing and cluster-free strains, PAA addition caused a small reduction of the biomass yield, consistent with PAA acting as a weak-organic-acid uncoupler. Microarray-based analysis on chemostat cultures of the high-producing and cluster-free strains, grown in the presence and absence of PAA, showed that: (i) Absence of a penicillin gene cluster resulted in transcriptional upregulation of a gene cluster putatively involved in production of the secondary metabolite aristolochene and its derivatives, (ii) The homogentisate pathway for PAA catabolism is strongly transcriptionally upregulated in PAA-supplemented cultures (iii) Several genes involved in nitrogen and sulfur metabolism were transcriptionally upregulated under penicillinG producing conditions only, suggesting a drain of amino-acid precursor pools. Furthermore, the number of candidate genes for penicillin transporters was strongly reduced, thus enabling a focusing of functional analysis studies.

**Conclusion:**

This study demonstrates the usefulness of combinatorial transcriptome analysis in chemostat cultures to dissect effects of biological and process parameters on gene expression regulation. This study provides for the first time clear-cut target genes for metabolic engineering, beyond the three genes of the β-lactam pathway.

## Background

Since the discovery of the production of antibiotics by the filamentous fungus *Penicillium chrysogenum *by Fleming in 1929 [1], much effort has been invested in selection and synthesis of strains with improved productivity [2,3]. This research has made decisive contributions to the successful large-scale production of β-lactam antibiotics after World War II. After isolation of the high-producing wild-type *P. chrysogenum *strain NRRL 1951 from a cantaloupe [4], random mutagenesis with irradiation or chemicals, followed by selection for superior production strains, enabled an over 1000-fold increase of penicillin productivity [5].

The penicillin biosynthesis pathway has been well characterized both genetically and biochemically. Biosynthesis starts with the condensation of the three amino acids cysteine, valine and α-aminoadipic acid to form the tripeptide ACV. This reaction is catalyzed by the non-ribosomal peptide synthase ACVS encoded by *pcbAB *[6-8]. In the next step, the classic β-lactam ring structure is formed by isopenicillinN synthase (*pcbC*, IPNS) [9]. IsopenicillinN forms the branch point for the penicillins and cephalosporins. Penicillins can be easily produced from isopenicillinN by the exchange of the α-aminoadipic acid moiety for a CoA activated side-chain, such as phenylacetic acid or phenoxyacetic acid, by acyl-CoA: isopenicillinN acyltransferase (*penDE*), which results in the production of penicillinG or penicillinV [6,7]. These three biosynthesis genes were shown to be physically linked in a penicillin biosynthesis gene cluster (*pcbAB-pcbC-penDE*) [7,10-13]. Present as a single copy in early strains, this gene cluster was shown to be present as amplified tandem repeats in later, high-producing strains [14,15]. Although increases in copy number have indeed been shown to result in improved productivity, saturation occurs at very high copy numbers [5,15], presumably due to limitations elsewhere in metabolism. In *P. chrysogenum *the biosynthesis pathway is compartmentalised. The enzymes involved in the first steps of biosynthesis, ACVS and IPNS, are localised in the cytosol, whereas the final steps, acyltransferase and the activation of the side-chain precursor by phenylacetyl-CoA ligase, take place in peroxisomes [16].

With some exceptions (e.g. clear increases in the copy numbers of penicillinG biosynthesis genes [14]), the molecular basis for high-level β-lactam production remains to be elucidated. Several cellular processes have been implicated in improved productivity, including a better utilisation of precursors, higher expression of biosynthesis genes, higher gene dosage and mutations in the regulatory genes controlling gene expression or other steps of the biosynthesis [12]. For rational and successful metabolic engineering, identification of these mutations will be of extreme benefit. With the availability of the *P. chrysogenum *genome sequence [17], it is now possible to study penicillinG production at a genome-wide scale.

PenicillinG production requires an exchange of the α-aminoadipic acid side-chain of isopenicillinN for the side-chain precursor phenylacetic acid (PAA) [18]. PAA is a weak acid (pK_a _= 4.3) and as such is likely to be toxic to cells depending on its concentration and the culture pH. Such toxicity may involve specific inhibitory effects of PAA on key enzymes in biomass or penicillinG production or, alternatively, more general mechanisms such as weak-acid uncoupling [19,20]. PenicillinG producing *P. chrysogenum *can metabolise PAA via at least two routes: incorporation in the penicillinG molecule or catabolism via the homogentisate pathway to acetoacetate and fumarate [21-24]. Although this catabolic route has been described in *P. chrysogenum *[25,26] little is known about the pleiotropic effects of PAA on *P. chrysogenum*. Only a few studies dedicated to the uptake of PAA have been reported and these show contrasting results. Hillenga *et al*. [20] and Eriksen *et al*. [27] both suggest that in production strains, and at moderate concentrations of PAA, this side-chain precursor enters the cell by simple diffusion across the plasma membrane. In contrast, at low external PAA concentrations, a high-affinity transporter has been proposed to contribute to PAA uptake by the Wisconsin54-1255 strain [27,28].

The specific rate of β-lactam production by *P. chrysogenum *is strongly dependent on the availability of a suitable side-chain precursor. In many studies on the physiological impact of β-lactam production, a comparison is made between cultures grown in the absence and presence of a side-chain precursor (e.g. phenylacetic acid, phenoxyacetic acid or adipic acid) [19,29,30]. While this approach has contributed to our insight in the energetics and kinetics of penicillinG production, it does not allow a clear distinction between effects caused by, on one hand, β-lactam production and, on the other hand, PAA consumption and/or toxicity. A suitable experimental approach would be to compare two strains with similar backgrounds under well-defined culture conditions by using chemostat cultures, in which one strain would only be lacking the penicillin biosynthesis cluster.

Mutants of *P. chrysogenum *impaired in penicillin biosynthesis have been described [31-33]. Most of these mutants were derived by random mutagenesis from the low-producing strain Wisconsin54-1255, which contains one copy of the penicillin biosynthesis cluster. Although these mutants were very useful for studying gene expression and gene/enzyme relationships, for identification of the factors important for penicillinG production and PAA consumption a strain obtained from a high-producing strain background by targeted deletion is more beneficial. To this end we constructed a strain in which the tandem-repeated penicillin biosynthesis cluster was specifically deleted, whereas the strain background was retained. In such a way it is possible to identify, by a combinatorial approach, those genes affected by phenylacetic acid and those specifically important for penicillinG biosynthesis.

## Results and Discussion

### Generation of a penicillin gene cluster-free derivative of a penicillinG high-producing *Penicillium chrysogenum *strain

**Figure 1:**
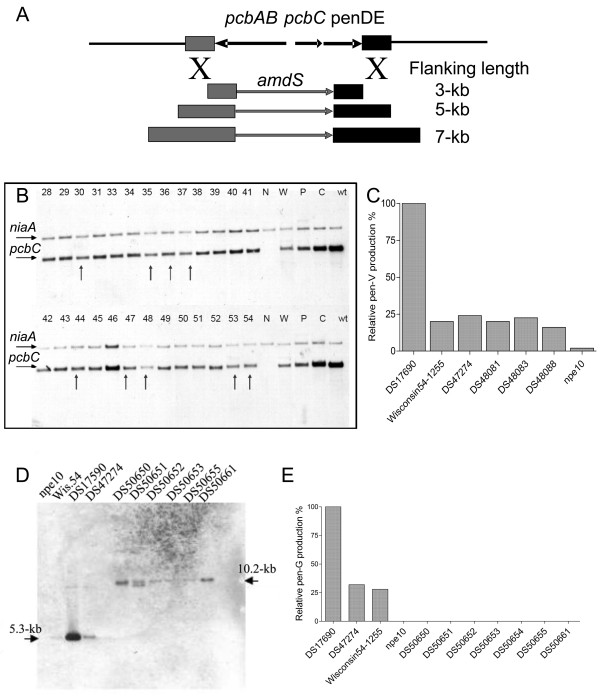
**Obtaining a penicillin-biosynthesis-gene-cluster-free strain**. **A**: Double homologous recombination strategy to delete the final biosynthesis gene cluster. A denotes the *pcbAB *gene, B the *pcbC *and *penDE *genes and M the marker gene *amdS*. **B**: Southern blot analysis to determine relative gene-copy number. Arrows indicate putative 'single copy' penicillin biosynthetic gene cluster candidates. Each number represents a single mutant. N, the non-producing isolate npe10; W, the lab strain Wisconsin54-1255, P, the parent strain DS47274, C, strain DS47276 with ± 8 copies and wt, the high-producing strain DS17690. **C**: Relative penicillinV production by putative single copy isolates (DS47274; DS48081; DS48083; DS48088) in shake flasks. **D**: Southern blot analysis to characterize the cluster free strain. Genomic DNA digested with *Hin*dIII was probed with a 425-bp fragment targeting the 3'*pcbAB *flanking region, cluster containing strain exhibited a band at 5.3-kb while cluster free strain exhibited a band of 10.2-kb resulting from the deletion of the last copy of the cluster. **E**: Relative penicillinG production by putative zero-amplicon mutants (DS50650–DS50671) in shake flasks.

To enable studies on the genome-wide impact of penicillinG biosynthesis in *Penicillium chrysogenum*, penicillin gene clusters (*pcbAB-pcbC-penDE*) were removed from the penicillinG high-producing strain *P. chrysogenum *DS17690. Since the β-lactam gene amplifications are in direct repeats of a larger amplified region on the same chromosome [14] spontaneous recombinations between different repeats can result in loss of gene cluster copies [15]. Isolates that underwent spontaneous recombinations were obtained via protoplast formation and subsequent sporulation of regenerating colonies. 27 random isolates were selected for Southern analysis to estimate the relative gene copy numbers of the penicillin biosynthesis gene *pcbC *and the single-copy *niaA *gene (Figure 1). The same strains were tested for penicillinV production in shake-flask cultures grown on mineral medium with the side-chain precursor phenoxyacetic acid. Half of the random isolates tested had both reduced *pcbC:niaA *ratio's and reduced penicillinV titres. One selected strain was subjected to a second round of protoplast formation and screening. Again, 27 random isolates were selected and analysed (Figure 1B). Several putative single-copy penicillin gene cluster candidates (indicated by arrows in Figure 1B) were identified based on a similar *pcbC:niaA *ratio to that of the single-copy Wisconsin54-1255 strain [7,14,15,34]. Three of these strains were tested in for penicillinV production, with the parental *P. chrysogenum *DS17690 parent, the intermediate parent DS47274, the laboratory strain Wisconsin54-1255 and a non-producing isolate of the latter strain, npe10 [31], as controls. All three isolates showed a drastically reduced penicillinV titre, comparable to that of the single-copy strain Wisconsin54-1255. It was therefore assumed that these three isolates are derivatives of *P. chrysogenum *DS17690 that carry a single copy of the penicillin biosynthesis gene cluster (Figure 1C). To delete the last remaining copy of the penicillin gene cluster, a double homologous recombination strategy was applied. Sequences adjacent to the β-lactam biosynthetic gene cluster (3' from the *pcbAB *gene and 3' from the *penDE *gene) were used as homologous flanking sequences to target the *amdS *selection marker to this locus and to delete the entire 17-kb region containing the cluster of β-lactam biosynthetic genes (*pcbAB-pcbC-penDE*) in the same integration event. Homologous integration by double crossover will generate transformants able to use acetamide as the sole nitrogen source (due to the presence of the *amdS *gene) but unable to produce any penicillins. Moreover, genomic DNA of strains that have undergone a successful double crossover should not hybridise to β-lactam gene specific probes. Of 27,076 transformants tested, 22 (0.08%) gave no inhibition zone when overlaid with a penicillin sensitive *E. coli *strain and were selected for further analyses. All 22 putative mutants were negative in the diagnostic PCR for the β-lactam biosynthetic gene *penDE*. Two of these strains gave no signal for *amdS*, suggesting that they had spontaneously lost the marker gene. Southern analysis of the obtained *P. chrysogenum *strains was subsequently carried out. As a probe, a DNA fragment of 425-bp from the *pcbAB *3'-flanking sequence was PCR amplified (Table 1, primers 19 and 20). All strains with intact penicillin biosynthesis gene sequences (*i.e*. the industrial parent strain as well as the single-copy copy strain) showed a 5.3-kb band in case of digestion with *Hin*dIII while putative β-lactam cluster-free DS17690 derivatives showed a 10.2-kb band (Figure 1D). To confirm their inability to biosynthesize β-lactams all 22 mutants were inoculated in liquid mineral medium with phenylacetic acid as precursor. Indeed, none of the mutants was capable of producing penicillinG (Figure 1E). It was concluded that all 22 strains are derivatives of the *P. chrysogenum *DS17690 strain in which all copies of the penicillin gene cluster have been deleted. Isolate DS50661 was used as cluster-free strain in this study.

**Table 1:** Strain construction

**ID primer**	**Gene**	**Direction**	**Sequence**
1	*pcbC*	FWD	GAT TGG CGC TCC TCG TTC ACC
2	*pcbC*	REV	CCA TTA TTT TTC TAG TCG ACA TGG CAT CGA TTC CCA AGG CCA ATG TCC CC
5	*LF 7-kb*	FWD	GTT ACA CGC TTT GAT TCT GTG **GGT ACC **GAT GTT ATA TTC AGC TAC
6	*LF*	REV	CCC AAT A**GC GGC CGC **AGT TGA TAA TAT CAA TAT CTA AAA CTC CC
7	*LF 5-kb*	FWD	GGC ATA TAC GAG CAT **GGT ACC **AGG GAC AGA TGC CCA TCC TTG
8	*LF 3-kb*	FWD	GTA TAA AAG GGG AG**G GTA CC**G GGA AAG ATT TGT GGG CCT G
9	*RF*	FWD	GTA TGT AGC T**GC GGC CGC C**TC CGT CTT CAC TTC TTC GCC CGC ACT
10	*RF 3-kb*	REV	CCG CCT TCC TCA CTA ACC GGC CGG CA**G GTA CC**G ATG GAC TCA GCA TTA TC
11	*RF 5-kb*	REV	CTC TAG AAT GCT ACG GCC GTT CGA **GGT ACC **TTA TAG GAA AAA GGT AG
12	*RF 7-kb*	REV	CCT TTT CGC TGA GCG GCC GCA ATC ACA **GGT ACC **GTT TTT GTC GTC
13	*amdS*	FWD	ATG CCT CAA TCC TGG GAA GAA CTG
14	*amdS*	REV	CTT GAC GTA GAA GAC GGC ACC GGC
15	*penDE*	FWD	CCC GCA GCA CAT ATG CTT CAC ATC CTC TGT CAA GGC
16	*penDE*	REV	ATG ACA AAC ATC TCA TCA GGG
17	*niaA*	FWD	CAC AGA GAA TGT GCC GTT TCT TTG G
18	*niaA*	REV	TCA CAT ATC CCC TAC TCC CGA GCC
19	*pcbAB*	FWD	GAA GAC GTC ATA CTT ATT CTC TG
20	*pcbAB*	REV	CGG CAT CGG ATA AAG AGA TCT GG

Primers used in this study (restriction sites are in bold). LF and RF, Left and Right flanking region respectively, location of the flanking region relative to the *amdS*-cassette (see Figure 1A). Size of the flanking region in kb. ID primer, uniquely numbered identifier of the primer used.

### Physiology of a penicillinG high-producing strain and a cluster-free derivative in chemostat cultures

The high-producing DS17690 strain and the cluster-free strain (DS50661) were grown in aerobic, glucose-limited chemostat cultures at a dilution rate of 0.03 h^-1^, in the presence and absence of the penicillinG side-chain precursor PAA. For each combination of strain and PAA presence or absence, at least three independent chemostat cultures were analysed. In the absence of PAA, no penicillinG was produced by the DS17690 strain; however intermediates such as isopenicillinN were still produced [35]. Under the same conditions, the cluster-free strain did not produce any β-lactam intermediates. The small difference between the biomass yields cultures of the high-producing and cluster-free strains grown in the presence of PAA confirms an earlier report [36] that penicillinG biosynthesis imposes an energetic burden on *P. chrysogenum*. However, the PAA-induced reduction of the biomass yield of the cluster-free strain (Table 2) suggests that part of the biomass yield decrease that is observed upon induction of penicillinG production by PAA addition [36], may in fact be caused by the side-chain-precursor PAA itself, e.g. via uncoupling of the plasma membrane [19,20].

**Table 2:** Physiological and microarray quality parameters for aerobic, glucose-limited chemostat cultures grown at a dilution rate of 0.03 h^-1^

	**DS17690 - PAA**	**DS17690 + PAA**	**DS50661 - PAA**	**DS50661 + PAA**
**Y_sx_^a ^(g·g^-1^)**	0.37 ± 0.01	0.35 ± 0.01	0.39 ± 0.01	0.36 ± 0.01
**q_pen_^b ^(μmol·g^-1^·h^-1^)**	0.00 ± 0.00	19.81 ± 1.47	0.00 ± 0.00	0.00 ± 0.00
**q_PAA_^c ^(μmol·g^-1^·h^-1^)**	0.00 ± 0.00	24.04 ± 2.38	0.00 ± 0.00	5.60 ± 0.38
**q_CO2_^d ^(mmol·g^-1^·h^-1^)**	1.15 ± 0.08	1.42 ± 0.11	1.16 ± 0.05	1.44 ± 0.10
**q_O2_^e ^(mmol·g^-1^·h^-1^)**	1.19 ± 0.11	1.42 ± 0.17	1.16 ± 0.11	1.43 ± 0.08
**Avg CV^f^**	0.21	0.18	0.17	0.14
**Pc actA^g^**	4190 ± 170	3560 ± 360	3450 ± 390	3030 ± 680
**Pc gdh2^h^**	1240 ± 120	1140 ± 270	1020 ± 170	1060 ± 180
**Repeats (n)**	3	4	3	3

*P. chrysogenum *strains DS17690 (penicillinG high producing) and DS50661 (lacking a functional penicillin gene cluster) were grown in the presence and absence of phenylacetic acid (PAA) in independent glucose-chemostat cultures at D = 0.03 h^-1^. Results are the averages ± S.D. (σ_n-1_).

^a ^biomass yield on glucose (g of biomass per g of glucose consumed)

^b ^biomass specific penicillinG production rate. In addition to penicillinG, intermediates and byproducts are formed, which accounts for ~6% of the consumed PAA [30] (μmol of penicillin G produced per g of dry weight biomass and per · hour)

^c ^Biomass specific phenylacetate consumption rate (μmol of phenylacetate consumed per g of dry weight biomass and per hour)

^d ^Biomass specific CO_2 _production rate (mmol of CO_2 _produced per g of dry weight biomass and per hour)

^e ^Biomass specific O_2 _consumption rate (mmol of O_2 _consumed per g of dry weight biomass and per hour)

^f ^the average coefficient of variation (standard deviation divided by the mean) for all genes except the genes with mean below 12

^g ^encoding actin; average hybridization signal and standard deviation

^h ^encoding glutamate dehydrogenase (NAD^+ ^dependent); average hybridization signal and standard deviation

Although PAA could not be used for penicillinG production in the cluster-free strain, it was still consumed at circa 25% of the rate observed in the DS17690 strain. The PAA consumption rate in the cluster-free strain corresponded quantitatively to the PAA consumption that was not incorporated into penicillinG in the high-producing strain (Table 2). As reported previously for industrial strains of *P. chrysogenum*, this could be the result of oxidation to 2-hydroxyphenylacetic acid by phenylacetate hydroxylase and subsequent catabolism via the homogentisate pathway [25,27].

### Experimental design of transcriptome analysis and global gene expression responses to penicillinG production and PAA consumption

**Figure 2:**
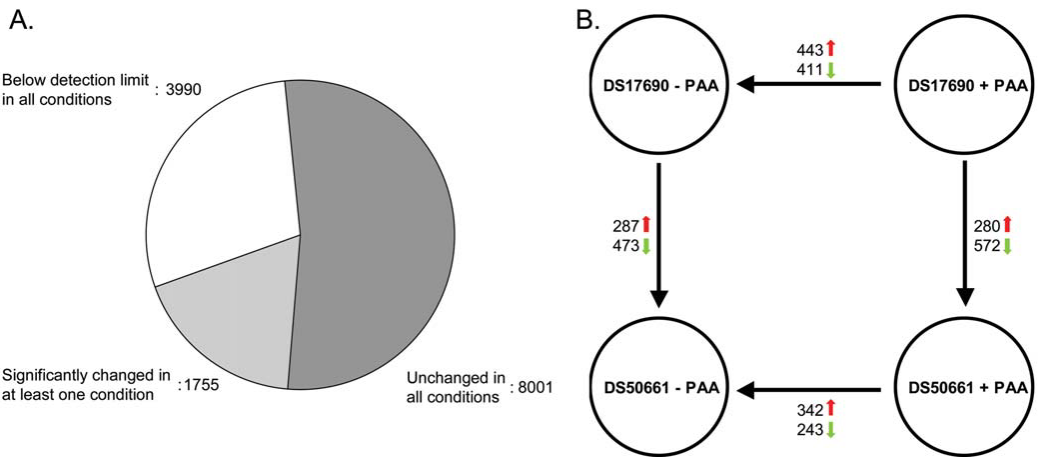
**Global gene expression response of DS17690 and DS50661 strains to the presence and absence of PAA**. Total RNA was obtained from *P. chrysogenum *strains DS17690 and DS50661, grown in the presence and absence of phenylacetic acid (PAA) in independent glucose-limited chemostat cultures at D = 0.03 h^-1 ^and hybridized to Affymetrix GeneChip^® ^microarrays. **A**: Pie chart of overall transcript differences of the DS50661 and DS17690 strains grown in the absence and presence of PAA. **B**: Results of the pairwise comparisons of the two strains and the two conditions. Red arrows indicate genes with a higher transcript level in the respective pairwise comparison, green arrows indicate genes with a lower transcript level.

Genome-wide transcriptome analysis was carried out on mycelium of both strains grown in glucose-limited chemostat cultures in the presence and absence of PAA at a dilution rate of 0.03 h^-1^. The average coefficient of variation of the transcriptome data derived from independent triplicate cultures did not exceed 0.21 (Table 2), which is similar to the reproducibility obtained with chemostat cultures of the non-filamentous yeast *Saccharomyces cerevisiae *[37]. The level of the *actA *[38] and *gdh2 *transcripts, which are commonly used loading standards for .Northern analysis, varied by less than 14% over the four situations tested. Chemostat experiments were designed to dissect gene expression responses to PAA and penicillinG production. Addition of PAA to growing DS17690 strain may induce two types of responses. Firstly, the presence of PAA itself may affect cellular processes outside β-lactam biosynthesis and thus affect the transcriptome of the fungus. Secondly, availability of a side-chain precursor enables the penicillinG formation, thus resulting in the induction and/or repression of the transcription of genes that are (in)directly related to β-lactam production. To dissect these two types of responses, we used the cluster-free strain DS50661 as a filter for the PAA response, as this strain neither produces penicillinG nor intermediates. Comparison of the transcript data of the two strains grown under the same conditions (DS17690+PAA versus DS50661+PAA; DS17690-PAA versus DS50661-PAA) will provide information on the effect of the removal of the penicillin cluster (and of possible unintended genetic changes resulting from the gene-cluster removal procedure) (Figure 2).

In total, four pair-wise comparisons between the two strains and the two conditions were performed (Figure 2), yielding a total of 1755 transcripts (representing 13% of the *P. chrysogenum *genome) that were differentially expressed in at least one of the comparisons based on the statistical criteria applied in this study (|fold difference| ≥ 2; false discovery rate 1%, see Methods section). The majority of the genome (8001 transcripts) did not show significant changes between the four conditions and transcript levels of 30% of the genome (3990 ORFs) was below the detection limit in all four situations (Figure 2).

**Figure 3:**
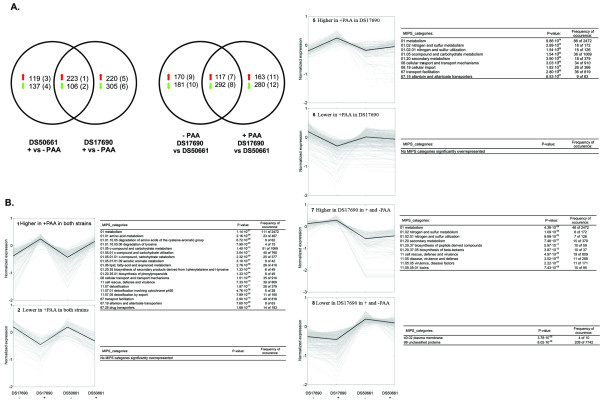
**Cross-sections, profiles and overrepresented functional categories of the pair-wise comparisons**. Transcript data from independent chemostat cultures of *P. chrysogenum *strains DS17690 and DS50661 grown at D = 0.03 h^-1 ^in the presence and absence of phenylacetic acid (PAA) were compared in four pairwise comparisons (DS17690 + PAA versus DS17690 - PAA; DS50661 + PAA versus DS50661 - PAA; DS17690 + PAA versus DS50661 + PAA and DS17690 - PAA versus DS50661 - PAA. Genes whose transcript level was significantly different in at least one of the four pairwise comparisons were overlapped as shown in **A**, resulting in 12 different groups of genes: 1–6: compare the response to PAA in DS17690 and DS50661; 7–12 compare the effect of the cluster removal. **B **shows the gene-transcript profiles of the groups of specific interest with the results of the hypergeometric distribution analysis for enrichment of functional categories. The thick line represents the average of the mean normalized transcript data of the genes comprising the cluster. The y-axis represents ^10^log transcript values. Functional categories are mentioned together with their P-value and the number of genes in the respective functional category in the group of genes with a higher transcript level compared to the prevalence of this functional category in the whole genome. Due to a large redundancy in the functional categories some categories might appear without having a significant biological relevance.

The set of differentially expressed genes was distributed over 12 groups following a two-way comparison (Figure 3) [see also Additional file 1]. Groups 1 and 2 (Figure 3) contained genes whose transcript levels were consistently higher or lower in the presence of PAA, irrespective of the strain background. Similarly, groups 7 and 8 harboured genes whose transcript levels were consistently higher or lower in the DS17690 strain, irrespective of the presence of PAA (Figure 3). Groups 5 and 6 represent genes that show a higher and a lower transcript level in the presence of PAA, but only in the DS17690 strain. These latter profiles are consistent with genes whose transcription is responsive to the production of penicillinG.

Enrichment of functional categories in these clusters was assessed according to the MIPS functional categories annotation [17,39] (Figure 3). To identify possible regulatory networks, a systematic search for possible protein-binding motifs in promoter sequences was performed on the different clusters.

### Gene expression responses to removal of the penicillin biosynthesis gene cluster

**Figure 4:**
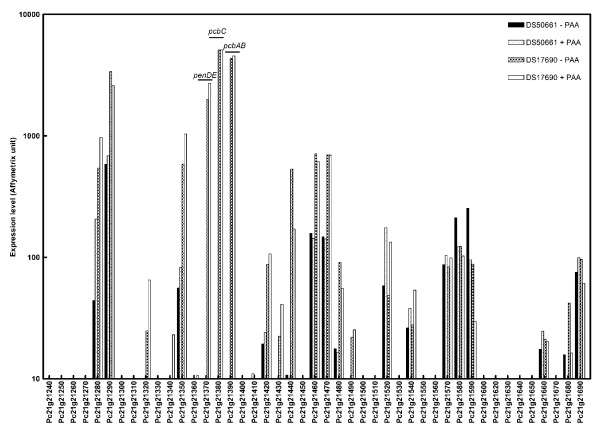
**Transcript profiles of the amplified region in penicillinG-producing strains of *Penicillium chrysogenum***. Transcript level of the penicillin biosynthesis genes embedded in a region that is present in tandem repeats in penicillinG-high-producing strains, the amplified region (Pc21g21280–Pc21g21420, [17,34,40]). Total RNA was obtained from *P. chrysogenum *strains DS17690 and DS50661, grown in the presence and absence of phenylacetic acid (PAA) in independent glucose-chemostat cultures at D = 0.03 h^-1 ^and hybridized to Affymetrix GeneChip^® ^microarrays.

A total number of 409 genes (117 in group 7 and 292 in group 8; Figure 3) showed significantly different transcript levels in cultures of the high-producing and cluster-free strains, irrespective of the presence of the side-chain precursor PAA. As expected, transcripts of the three biosynthesis genes, *pcbAB, pcbC *and *penDE *could not be detected in the cluster-free strain. As a result of the strain construction the other genes in the amplified region (Pc21g21280–Pc21g21420) are present as a single copy in the DS50661 strain. The observation that several of these genes showed reduced expression levels in the DS50661 strain would therefore be consistent with a gene-dosage effect (Figure 4). In addition, as confirmed by studies on a different industrial strain of *P. chrysogenum *[40] and on the laboratory strain Wisconsin54-1255 [34], not all genes in the amplified region are transcriptionally induced under penicillinG producing conditions. In addition to an enrichment of functional categories related to β-lactam biosynthesis (01.20; 01.20.37.05; 11; 11.05, 11.05.05; and 11.05.05.01), group 7 (increased transcript levels in the high-producing strain DS17690) was also enriched for functional categories 01.02 and 01.02.01, which are related to nitrogen and sulfur metabolism (Figure 3). Upregulation of the synthesis of (sulfur-containing) amino acids may be indicative for an increased synthesis of the amino-acid β-lactam precursors. The observation that higher transcript levels of these genes were also observed in the DS17690 strain when it was grown in the absence of the side-chain precursor PAA may indicate that even the low net production of β-lactam intermediates under these conditions has an impact on mRNA level regulation of precursor biosynthesis.

**Figure 5:**
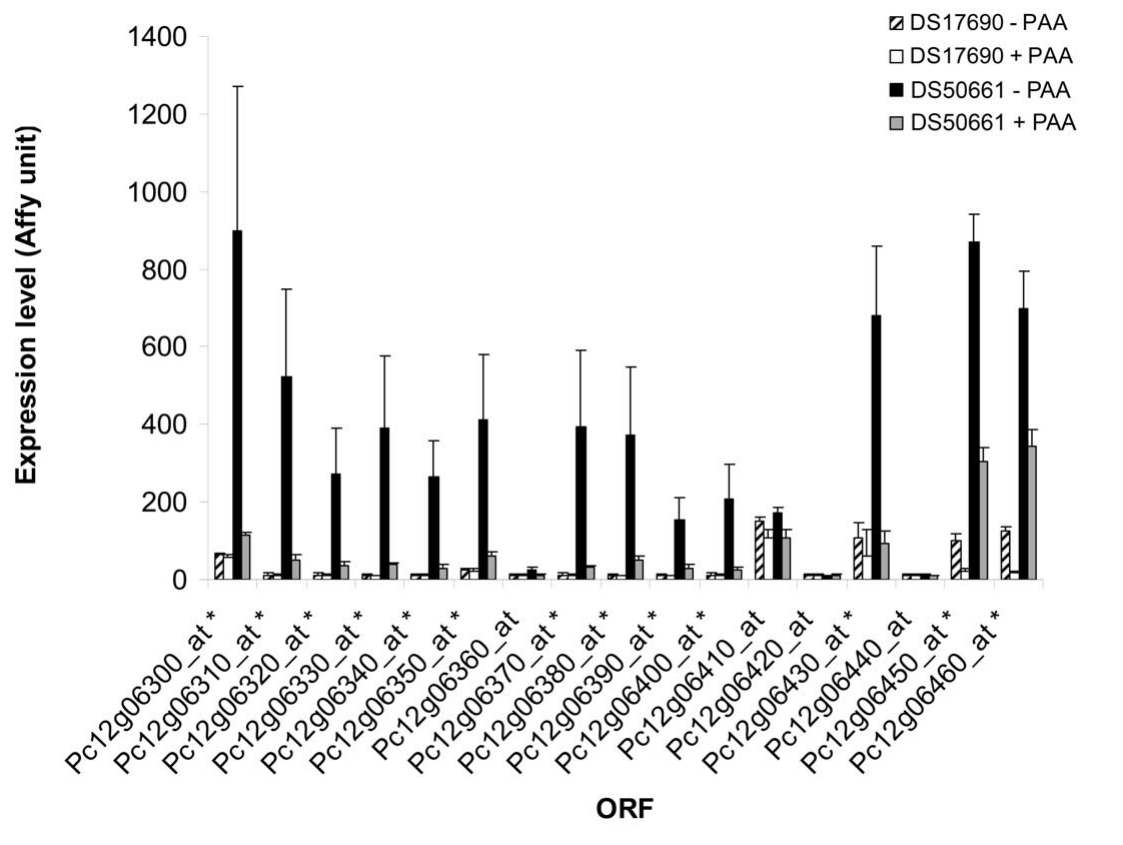
**Clustered genes with a higher transcript level in the DS50661**. Transcript levels of a cluster of genes of which many exclusively had higher transcript levels in the cluster-free strain DS50661 than in the penicillin-high-producing strain DS17690 irrespective of the side chain precursor. Annotation of many of these genes and their clustering suggests a role in secondary metabolite production. Total RNA was obtained from *P. chrysogenum *strains DS17690 and DS50661, grown in the presence and absence of phenylacetic acid (PAA) in independent glucose-chemostat cultures at D = 0.03 h^-1 ^and hybridized to Affymetrix GeneChip^® ^microarrays. *, genes with a significantly higher transcript level (fold change > 2, FDR 1%) in DS50661 compared to DS17690.

Among the 292 genes that were transcribed at higher levels in the penicillin-biosynthesis gene cluster-free strain than in the DS17690 strain (group 8) 17 genes were physically linked in the same chromosomal region (Figure 5). Although the increased transcript level of these genes was also observed in the presence of PAA, it was most pronounced in its absence. Annotation of many of these genes and their clustering suggests a role in secondary metabolite production (Figure 5) [41]. Among those 17 genes, the paralog of the aristolochene synthase gene, *Ari1*, from *Penicillium roqueforti *[42], which shares 97% identity, was identified (Pc12g06310). Synthesis of aristolochene represents the first step in the synthesis of sesquiterpenoids, which include potent anticancer compounds [43,44]. In *Penicillium roqueforti*, aristolochene is a precursor of PR toxin [45], for which the further biosynthesis pathway remains unidentified.

### Gene expression responses to the side-chain precursor phenylacetic acid (PAA)

**Figure 6:**
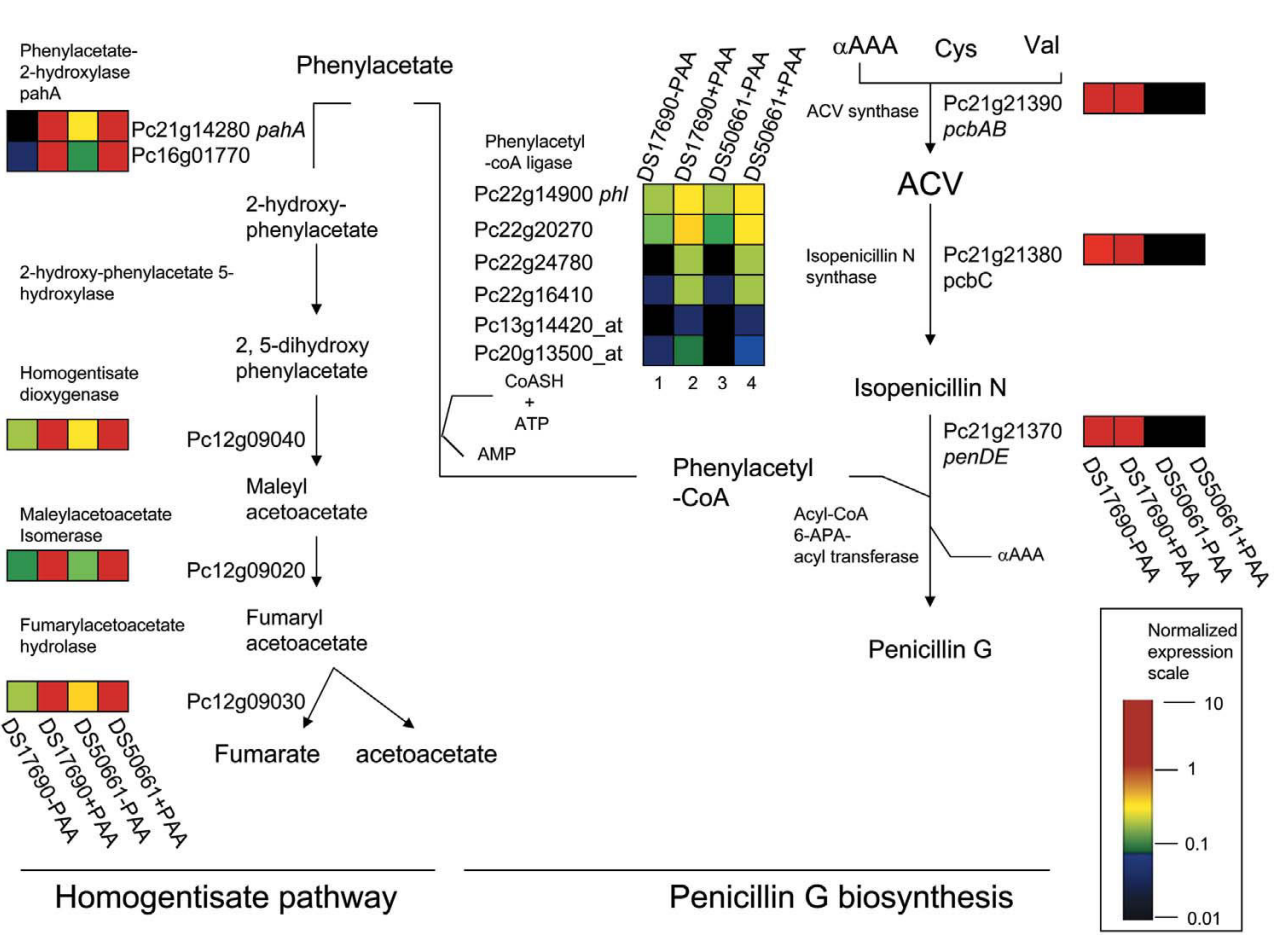
**Detoxification of phenylacetic acid via incorporation into penicillinG or the homogentisate pathway**. Those genes (putatively) related to penicillin, biosynthesis and phenylacetate catabolism are depicted. Total RNA was obtained from *P. chrysogenum *strains DS17690 and DS50661, grown in the presence and absence of phenylacetic acid (PAA) in independent glucose-chemostat cultures at D = 0.03 h^-1 ^and hybridized to Affymetrix GeneChip^® ^microarrays. The color bar indicates the range of the mean normalized transcript level value per gene. αAAA: α-aminoadipate, Cys: cysteine and Val: valine.

329 genes (groups 1 and 2, Figure 3) showed a consistently different transcript level in the presence and absence of PAA. Remarkably, genes belonging to the penicillin biosynthesis gene cluster were not differentially expressed in the presence and absence of PAA, with the *pcb *genes still being highly expressed in the absence of PAA. Only the gene encoding the PAA CoA ligase (*phl*, Pc22g14900) [46] showed a significantly higher transcript level in the presence of PAA (increase of 70 and 100% relative to cultures lacking PAA for the DS17690 and DS50661 strains, respectively). Recent functional characterization of *phl *revealed that upon its deletion, the PAA-CoA ligase activity decreased by only 40% [46], indicating the existence of one or more additional PAA-CoA ligases. Five genes with similarity to aryl- or fatty-acid CoA ligases were expressed to a higher level in cultures of both strains grown in the presence of PAA. The two closest *phl *homologues (Pc22g24780 and Pc22g20270) showed over 65% identity with *Aspergillus *proteins of unknown function, as well as a strong similarity with *Arabidopsis thaliana *4-coumarate-CoA ligase (30%) (Figure 6). Further inspection of the predicted amino acid sequences of both these genes identified the presence of PTS1 peroxisomal targeting sequences. Based on these observations, Pc22g24780 and Pc22g20270 represent interesting candidates for further functional analysis aimed at identifying alternative PAA CoA-ligases in *phlΔ *strains.

Both strains showed penicillinG independent metabolism of PAA. In the DS17690 strain, this was evident from the observation that PAA consumption exceeded penicillinG production (Table 2). Indeed, the PAA hydroxylase gene (*pahA*) [25] that encodes the first step of the PAA catabolism through the homogentisate pathway was highly induced in cultures grown in the presence of PAA (from +10- to over 100-fold). However, it has been reported, already early in the strain improvement lineage that a mutation (L181F) in this gene results in a dramatic reduction of enzyme activity [25]. Interestingly, a second gene (Pc16g01770), whose predicted protein sequence shared 42% identity with the *pahA *product also showed strongly elevated transcript levels (≥ + 20-fold) in the presence of PAA. This second gene is 82% identical to *Aspergillus nidulans PhacB *[23], which encodes a 3-hydroxyphenylacetate 6-hydroxylase and 3,4-dihydroxyphenylacetate 6-hydroxylase cytochrome P450 monooxygenase capable of converting PAA into 2-hydroxyphenylacetate. This second gene may well be responsible for residual PAA catabolism in industrial strains that carry a loss-of-function mutation in *pahA*. Based on genome annotation and the transcript profiles of cultures grown in the presence and absence of PAA, the entire homogentisate pathway, which ultimately leads to the formation of fumarate and acetoacetate, could be mapped. Genes encoding a homogentisate dioxygenase (Pc12g09040); a maleylacetoacetate isomerase (Pc12g09020) and a fumarylacetoacetase (Pc12g09030) were tentatively identified, completing the identification of the metabolic pathway. In contrast to the other PAA-utilizing pathway (penicillinG synthesis) the five genes of the homogentisate pathway all showed a strong gene level upregulation in presence of PAA (ranging from +6.6-fold to +90-fold). Apparently, despite the reduction of the pathway's activity, transcriptional regulation of the homogentisate pathway is still functional in high-producing strains of *P. chrysogenum*. As indicated by their gene identity codes, the ORFs Pc12g09020, Pc12g09030 and Pc12g09040 form a chromosomal cluster. A similar clustering of the homogentisate pathways genes has been observed in *Aspergillus nidulans *[47]. An additional gene of this cluster, Pc12g09010, that shares 51% of identity with *A. nidulans *AN1893.3 and displays similarity with a putative transcription factor from *Neosartorya fischeri*, was also upregulated in presence of PAA in both DS17690 and DS50661 (+2.6-fold and +3.8 respectively). This observation makes it tempting to speculate that Pc12g09010 participates in transcriptional regulation of PAA catabolism (Figure 6). The characterisation of the genes encoding the pathway (a homogentisate dioxygenase (Pc12g09040); a maleylacetoacetate isomerase (Pc12g09020) and a fumarylacetoacetase (Pc12g09030)), the physical clustering of these genes and the presence of a co-clustered putative transcription factor represent interesting targets for metabolic engineering to eliminate residual rates of PAA consumption and to alleviate a potential protein burden [48] imposed by high-level induction of this pathway.

Transport mechanisms for β-lactam antibiotics and side-chain precursors, both across the fungal plasma membrane and between intracellular compartments, remain incompletely understood. The functional category analysis of genes that showed an increased transcript level in cultures grown in the presence of PAA showed a clear enrichment of the transport-related functions (Figure 3). 42 genes in the functional categories 'cellular transport and transport mechanisms' and 'transport facilitation' showed a significantly increased transcript level in cultures grown with PAA in both strains (Figure 3, group 1). The uptake of undissociated phenylacetic acid in *P. chrysogenum *has been reported to occur via passive diffusion [20]. However, by analogy to the well-studied non-filamentous yeast *Saccharomyces cerevisiae*, in which PAA is exported by the ATP-binding cassette transporter Pdr12 [49], its anion form is likely to be actively exported into the medium as a detoxification mechanism. From the available functional annotation of the *Penicillium *genome, 2 out the 42 genes (Pc22g14600 and Pc22g20390) display motif signatures of ABC transporters, as well as sequence similarity to the *Aspergillus nidulans atrB *[50] and *atrD *[51] proteins, respectively. Only Pc22g14600 belongs to the ABC-G transporters cluster [17] that also includes Pdr12, which makes Pc22g14600 a very attractive candidate for further characterization.

Activation of PAA and the final biosynthetic step in the penicillinG biosynthesis pathway, the exchange of the aminoadipic acid side-chain for PAA, both occur in the peroxisome. This metabolic compartmentation of penicillinG production makes transport of isopenicillinN, PAA and penicillinG across the peroxisomal membrane an integral and important part of penicillinG biosynthesis. Only one of the transporter genes that showed an increased transcript level in cultures grown with PAA (Pc21g09430) showed a clear link with peroxisomes. This gene shows strong similarity to the *Saccharomyces cerevisiae ANT1 *gene that encodes a peroxisome-localised protein involved in adenine nucleotide transport, medium-chain fatty acid metabolism, and peroxisome proliferation [52].

The genes that showed a consistently lower transcript level in cultures grown in the presence of PAA (Figure 3 Group 2 (106 genes)), failed to show a clear enrichment of any functional category and, moreover, showed a high incidence of genes with unknown function and/or similarity with a gene of unknown function in another organism.

### Dissection of gene expression responses to PAA and to penicillinG production

For future studies into the mechanism, compartmentation and regulation of penicillinG biosynthesis, it would be helpful to dissect transcriptional responses of side-chain precursor availability and penicillinG biosynthesis itself. The cluster-free strain described above synthesizes neither penicillinG nor any of its intermediates, even when grown in presence of PAA. Consequently, genes that show a transcriptional response to PAA that is specific for the high-producing strain DS17690 are likely to be related to penicillinG production rather than to the presence of PAA per se (Groups 5 and 6, Figure 3).

**Figure 7:**
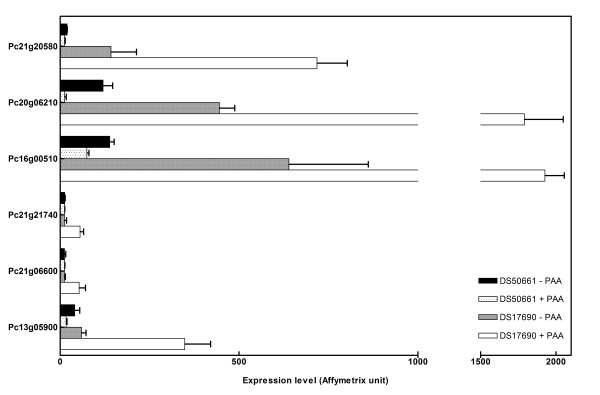
**Transcript levels of putative sulfonatase genes specifically responding to penicillinG production**. Group 5 containing genes exclusively responding to penicillinG biosynthesis contains six putative sulfonatases. Total RNA was obtained from *P. chrysogenum *strains DS17690 and DS50661, grown in the presence and absence of phenylacetic acid (PAA) in independent glucose-chemostat cultures at D = 0.03 h^-1 ^and hybridized to Affymetrix GeneChip^® ^microarrays.

This approach yielded 220 genes (Group 5, Figure 3) whose transcript level was specifically higher in penicillinG producing cultures of the DS17690 strains and 305 genes (Group 6, Figure 3) that showed a lower transcript level in these conditions. Group 5 was enriched for genes whose annotation involved functions in nitrogen and sulfur metabolism, secondary metabolism and transport. Among the 18 genes assigned to the nitrogen and sulfur metabolism FunCAT annotation, six were sharing significant homology with dioxygenases (sulfur dioxygenase, taurine dioxygenase, 2-oxoglutarate-dependent dioxygenase, arylsulfatase, Fe(II)-dependent sulfonate alpha-ketoglutarate dioxygenase, Figure 7). Although these enzymes participate in the mobilisation of sulfur from alternative sources (e.g., in the absence of sulfate [53]), the analogy with the low-sulfate response as recorded in *S. cerevisiae *[54,55] cannot be extended further. For example, consistent with the excess sulfate included in the growth media, we did not observe a transcriptional upregulation of key enzymes in sulfate assimilation (e.g. genes involved in methionine biosynthesis). However, the transcript level of the sulfate transporter gene *sutB *[56] was specifically increased by 60 % in the penicillinG producing cultures of the DS17690 strain. Moreover, the *sutB *transcript level was +4.4-fold higher in the DS17690 strain than in the DS50661 strain when grown in the presence of PAA. A similar transcript profile was observed for Pc18g03480, which has a strong similarity with high-affinity methionine permease genes. The second *P. chrysogenum *sulfate transporter gene *sutA *[56] was transcribed at very low levels under all conditions tested. Specific transcript level increase of several genes involved in sulfur and nitrogen assimilation in penicillinG producing scenarios suggests that a drain of amino acid precursors (cysteine and possibly valine) may affect intracellular pools of these amino acids. Indeed, metabolic flux analysis of *P. chrysogenum *grown under producing and non-producing conditions showed that the flux from 3-phosphoglycerate to serine and cysteine biosynthesis was 6-fold higher under penicillinG-producing conditions [30]. One gene involved in serine and cysteine synthesis (Pc21g03190, encoding a putative hydroxypyruvate dehydrogenase) showed an increased transcript level in penicillinG producing cultures of the DS17690 strain. This identifies the analysis, and possibly engineering, of cysteine and valine biosynthesis as relevant activities in applied research on β-lactam production.

The transporter for penicillinG in *P. chrysogenum *is still unknown. Although it cannot be excluded that the penicillin transporter is among the 700 constitutively transcribed transporters, the 36 transporters in group 5 form an interesting group for initial analysis. Out of the 36 transporter-encoding genes found in group 5, 18 were assigned to transport of a wide range of nitrogen sources, including urea (2 genes), allantoate (7 genes), various amino acids including lysine and methionine (8 genes) and oligopeptides (1 gene). Whereas lysine and methionine transport might be related to the sulfur status of the cells (see above) the role of the other genes is more elusive. However, two observations on transport-related genes provide interesting leads for follow-up studies. Firstly, Pc22g11250, whose transcript profile correlated perfectly with the production of penicillin G, shows strong similarity with the *A. niger *gene An15g07460, which encodes an oligopeptide transporter. Interestingly transport of β-lactams through human intestinal epithelium involves an oligopeptide (di- and tripeptide) transporter [57]. We are currently investigating the possibility that this transporter is involved in penicillinG export.

The second example *a priori *has no relationship with the penicillin synthesis; a group of 8 genes, whose products all show similarities with the yeast allantoate transporter Dal5, exhibited a significant upregulation under penicillinG producing conditions. These genes belong to a larger genome-wide family of 30 members. Although these 30 transporters share the same description "strong similarity to Dal5", they display very different expression profiles. Without functional analysis studies on these genes, any biological interpretation of this observation would remain speculative.

Along with the penicillinG synthesis, 18 genes that could be involved in secondary metabolism were also expressed to a higher level under penicillinG producing conditions. This group harboured two genes Pc21g23730 and Pc21g20650 that exhibit strong similarities with a feruoyl-CoA synthetase from *A niger *and a 4-coumarate-CoA ligase from *Arabidopsis thaliana*, respectively. While the transcript levels of these genes, remained lower than those of the two PAA-inducible putative aryl-CoA ligases mentioned above, this does not rule out a possible contribution of their gene products to *in vivo *PAA activation, which is further supported by the putative peroxisomal targeting signal that both harbour.

### Analysis of upstream regulatory sequences

**Figure 8:**
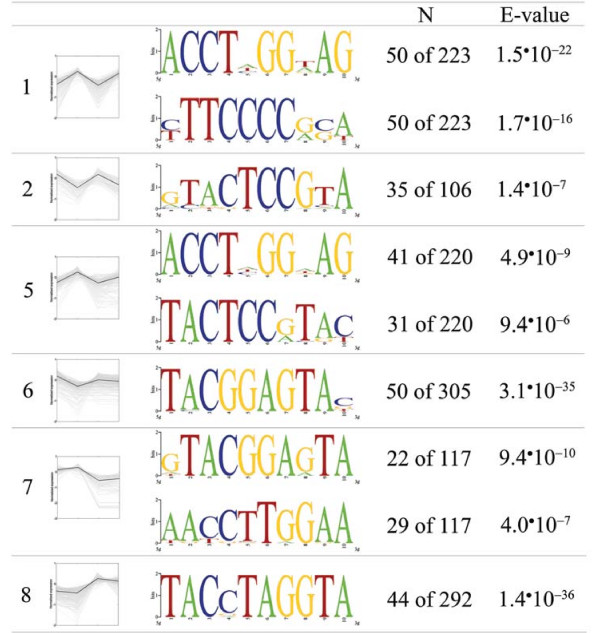
**Predominant motifs identified in 800 nucleotide upstream region**. Promoter analysis of the 800 nucleotide upstream region of sets of co-regulated genes, identified by overlapping the results of the four pairwise comparisons (groups 1, 2, 5, 6, 7, 8), using MEME. Motifs with an E-value < 10^-5 ^and without long stretches of A and T (> 40% GC content) were included in the analyses. Consensus sequences were depicted using the web based application WebLogo, version 2.8.2 [86,87]. N denotes the number of genes with the motif compared to the number of genes in the co-regulated group.

The 800 nucleotides upstream of the ATG of groups of genes that showed a similar transcriptional regulation were analysed for *cis*-regulatory elements. MEME analysis identified two motifs in group 1 and 1 motif in group 2 that met the statistical criteria applied (Figure 8; see Materials and Methods for details). Although these motifs had a good coverage of the genes in the group, the most strongly regulated genes, those involved in PAA catabolism, did not contain this motif in their 800 nucleotide upstream region. In addition, some of the motifs identified are shared by the different co-regulated groups and none match any of the known binding sites of the limited set of described transcription factors (Figure 8). Possibly the long history of strain improvement of these strains has resulted in a loss of conserved motifs. Nevertheless, a similar analysis performed on a set of 53 co-regulated genes responding to PAA in the lab strain Wis54-1255 and the DS17690 strain (cluster 1, described in [17]) identified the same motifs as found in the present study (motif 1 from group 1 and motif 2 from group 7). Increasing the length of the motif to be identified resulted in the same core conserved nucleotides (data not shown). In addition, as the groups of co-regulated genes are quite large, the discriminating power of MEME may not be sufficient to identify the actual conserved and regulating motifs. Testing a small subset of the genes, the 7 genes known to be directly involved in PAA catabolism, indeed resulted in different motifs that had a very high coverage over the genes tested. However, none of these motifs passed the stringent statistical criteria applied. These preliminary results suggest that motif identification in filamentous fungi may require different approaches than hitherto applied in more intensively studied microorganisms.

## Conclusion

80 years after Fleming's discovery of the antibacterial activity of penicillin, research on *Penicillium chrysogenum *has now become accessible to genomics approaches [17]. In the present study, we have integrated microarray-based transcriptome analysis with chemostat cultivation. This approach, which has already shown to be fruitful in other organisms such as *Saccharomyces cerevisiae *[54,55,58,59], *Trichoderma reesei *[60] and *Escherichia coli *[61,62], enables an investigation of the effect of individual culture parameters on genome-wide gene expression regulation. Reproducibility of transcript data is often cited as an additional advantage of chemostat-based microarray analysis [63]. Although steady-state chemostat cultivation of filamentous fungi is experimentally more challenging than chemostat cultivation of non-filamentous microorganisms, the excellent reproducibility of the transcript data obtained with *P. chrysogenum *indicates that this does not preclude accurate and reproducible chemostat-based transcriptome analysis.

In aerobic, glucose-limited chemostat cultures of *S. cerevisiae*, ca. 86 % of its 6400 genes [37] showed a detectable transcript level. Of the much larger genome of *P. chrysogenum*, cultivation under similar conditions yielded a detectable transcript for only 67 % of the genes. Furthermore, of the 1755 genes that showed a differential transcript level under at least one of the conditions, 53% has an unknown function. This percentage is similar to the percentage of unclassified proteins throughout the whole genome sequence [17]. These observations illustrate the formidable challenges that remain to be addressed in the functional analysis of the genomes of filamentous fungi. The identification of gene function in *P. chrysogenum *is likely to benefit tremendously from the rapid sequencing, annotation and analysis of the genomes of other filamentous fungi, such as those of *N. crassa *[64], *A. fumigatus *[65], *A. nidulans *[47], *A. oryzae *[66], *A. niger *[67,68], *T. reesei *[69]. For example, the recent characterisation of a new sulfate transporter gene, *astA*, in *A. nidulans*, homologous to the *S. cerevisiae *Dal5 transporter [70] enabled us to tentatively interpret the involvement of a similar gene in *P. chrysogenum *as being part of a broader sulfur-related response.

Carefully designed transcriptomics experiments can help to prioritize targets for functional analysis based on at least three criteria: (i) the absence of a detectable transcript level rules out that the gene product contributes to either fitness or industrial performance under the experimental conditions, (ii) gene expression regulation can provide insight into the possible role of gene products in an experimental context, although relations between transcript profiles and contribution to fitness are not necessarily straightforward [71-73], and (iii) the availability of possible sequence-derived information on the putative catalytic, structural or regulatory role(s) of gene products that suggest a role in fitness and/or industrial performance [63]. Based on these criteria, several priority targets for future functional analysis studies have been identified in the present study (see Results and Discussion section).

The present study demonstrates how a simple combinatorial design of chemostat experiments, involving two *P. chrysogenum *strains, can be applied to dissect effects of side-chain-precursor availability and β-lactam production. While similar approaches have previously been applied to dissect effects of oxygen availability and nutrient limitation in *S. cerevisiae *[54,55,74], this is to our knowledge the first application of such an approach to a product-forming system. Our experimental design required a strain of *P. chrysogenum *that lacked a functional penicillin gene cluster. A cluster-free strain previously described [32], was derived from the low-producing Wisconsin54-1255 strain. As we sought to maximize the difference between producing and non-producing scenarios, a new cluster-free strain (DS50661) was derived from a penicillinG-high-producing strain background (DS17690). The approach presented in this paper should be applicable to the production of other secondary metabolites in systems where production can be controlled by the addition of a precursor molecule. In *P. chrysogenum*, this might for example include the production, by engineered strains, of the cephalosporin precursors 7-amino-deacetoxycephalosporanic acid (7-ADCA) [75], adipoyl-7-amino-3-carbamoyloxymethyl-3-cephem-4-carboxylic acid (ad7-ACCCA) [76] and deacetylcephalosporin C [77].

## Methods

### Strains

*Penicillium chrysogenum *strains DS17690 is a high producing strain and derived from the strain improvement program of DSM [29,35]. DS50661 lacks the penicillin biosynthesis cluster and was constructed from DS17690 as described in this paper.

### Preparation of protoplasts

Preparation of *P. chrysogenum *protoplasts was performed as described by Cantoral *et al*. [78], using Glucanex (Novo Nordisk, Bagsvaerd, Denmark) instead of Novozyme as the cell wall degrading enzyme. Protoplasts were separated from the mycelium, washed and plated on mineral medium agar [79], without phenylacetic acid, but supplemented with 15 g.l^-1 ^agar to solidify and 1 M sucrose for osmotic stabilization. Regenerating colonies were transferred to plates without sucrose to induce sporulation. Spores were collected, washed with 0.85 % NaCl, diluted and plated out on YEPD agar plates (10 g l^-1 ^Yeast Extract, 10 g l^-1 ^Peptone, 20 g l^-1 ^glucose and 15 g l^-1 ^agar). Isolated colonies were transferred to mineral medium agar plates serving as stock culture plates.

### Genomic DNA isolation

To isolate genomic DNA, *P. chrysogenum *strains were grown in mineral-medium shake-flask cultures for 48 h at 25°C and 280 rpm. Cells were harvested, washed with 0.85 % NaCl and the pellet was frozen in liquid N_2_. Frozen cells were grinded using a pestle and mortar, transferred to a plastic tube and an equal volume of phenol:CHCl_3_:isoamylalcohol (25:24:1) was added. This mixture was vortexed vigorously, centrifuged and the aqueous phase was transferred to a fresh tube. This procedure was repeated twice, each time using a fresh volume of phenol:CHCl_3_:isoamylalcohol (25:24:1). Finally, DNA was isolated from the aqueous phase by ethanol precipitation according to standard procedures.

### Estimation of penicillin biosynthetic gene cluster copy numbers

Genomic DNA (3 μg) was digested with *Eco*RI, separated on a 0.6% agarose gel and transferred to a nylon membrane by vacuum Southern Blotting. Fragments of the *pcbC *and *niaA *genes were used as probes. The former probe gives an indication of the copy number of penicillin biosynthetic genes and the latter probe is a single copy gene (encoding nitrite reductase), in *P. chrysogenum *DS17690. The probe sequences were amplified using gene specific primers 1–4 (Table 1) and labelled with the ECL non-radioactive hybridisation kit (Amersham, Little Chalfont, UK) according to the supplier's instructions. The ratio between the intensity of both signals (*pcbC */*niaA*) was used to estimate the relative gene copy number of the penicillin-gene cluster. The parent strain DS17690 and the single-copy lab strain Wisconsin54-1255 were used as controls.

### Deletion of a single-copy penicillin biosynthetic gene cluster

To delete a single copy of the penicillin gene cluster that remained after spontaneous homologous recombination events (see Results section) a double homologous recombination strategy was applied. As double homologous crossover is a rare event in *P. chrysogenum*, three constructs were generated with 3, 5 and 7 kb homologous flanks on each side of the *amdS *gene respectively (Figure 1B). The oligonucleotides used are listed in Table 1. Following PCR amplification, the fragments were cloned in pCRXL (Invitrogen, Carlsbad, USA) via TOPO T/A cloning (Invitrogen). Subsequently, all three 5' flanking sequences (3, 5 and 7 kb) were digested with *Acc*65I and *Not*I followed by ligation in pBluescript II SK+ (Invitrogen) pre-digested with *Acc*651 and *Not*I (Table 3). The resulting plasmids carrying the 5'-flanking fragments were then digested with *Not*I to facilitate cloning of the 3' flanking sequences, which were pre-digested with *Not*I and *Eco*521 (Table 3). The obtained 3-, 5- and 7-kb flanking-plasmids all had a unique *Not*I site between the 5' and 3' flanking sequences, which was used to insert the *amdS *gene as selection marker. This was obtained by digesting pHELY-A1 [80] with *Not*I and isolating the 3.1-kb *PgpdA*-_An_*amdS *expression cassette. The *amdS*-containing deletion fragments were isolated after digestion with *Kpn*I and transformed to the single copy penicillin gene cluster strains. Transformants were selected for their ability to grow on acetamide-containing plates and afterwards screened for antibiotic production by replica plating colonies on mineral medium and overlaying them after 4 days of growth with the β-lactam sensitive indicator organism, *Escherichia coli *strain ESS2231 [81]. Isolates that did not show an inhibition zone were analysed via colony PCR with three primer sets: *amdS*, for the selection marker (primers 13 and 14); *penDE*, as indicator for the presence or absence of the penicillin biosynthetic gene cluster (primers 15 and 16) and *niaA *as an internal control (primers 17 and 18; Table 1). Finally, the complete removal of the three penicillin biosynthetic genes was confirmed by Southern blot. Genomics DNA (3 μg) was digested by *Hin*dIII, and probed with a 425-bp fragment corresponding to the *pcb*AB-3' flanking region amplified using the primers 19 and 20 (Table 1).

**Table 3:** Primer sets used for construction of double homologous crossover cassettes

		**Forward primer**		**Reverse primer**	
**Flank**	**Size (kb)**	**ID primer**	**Introduced restriction site**	**ID primer**	**Introduced restriction site**

Left	7	5	Acc*65I*	6	*Not*I
Left	5	7	*Acc*65I	6	*Not*I
Left	3	8	*Acc*65I	6	*Not*I
Right	3	9	Not*I*	10	Eco*52I*, Acc*65I*
Right	5	9	*Not*I	11	*Eco*52I, *Acc*65I
Right	7	9	*Not*I	12	*Eco*52I, *Acc*65I

### Media and media composition

The mineral medium was prepared as described [35] and contained per litre of demineralised water 7.5 g glucose, 3.5 g (NH_4_)_2_SO_4_, 0.8 g KH_2_PO_4_, 0.5 g MgSO_4_·7H_2_O, 10 mL of a trace element solution. The trace element solution contained 15 g·L^-1 ^Na_2_EDTA·2H_2_O, 0.5 g·L^-1 ^Cu_2_SO_4_·5H_2_O, 2 g·L^-1 ^ZnSO_4_·7H_2_O, 2 g·L^-1 ^MnSO_4_·H_2_O, 4 g·L^-1 ^FeSO_4_·7H_2_O, and 0.5 g·L^-1 ^CaCl_2_·2H_2_O. Production of penicillinG was induced by adding 0.58 g·L^-1 ^phenylacetic acid (PAA) to the medium. To ensure similar residual concentrations of PAA, 0.30 g·L^-1 ^was added to the medium of the cluster-free strain DS50661.

### Chemostat cultivation

Aerobic glucose-limited chemostat cultivation was performed at 25°C in 3-litre turbine-stirred bioreactors (Applikon, Schiedam, The Netherlands) with a working volume of 1.8 L. The pH was maintained at 6.5 via automated addition of 2 M NaOH (ADI 1030 biocontroller, Applikon, Schiedam, The Netherlands). The fermentor was sparged with air at a flow rate of 0.9 L·min^-1 ^using a Brooks mass-flow controller (Brooks Instruments, Hatfield, USA) and stirred at 750 rpm. The dissolved-oxygen concentration was continuously monitored with an oxygen electrode (Applisens, Schiedam, The Netherlands). Continuous cultivation was initiated after 50–60 h of batch cultivation. The feed medium was supplied continuously by a peristaltic pump (Masterflex, Cole Parmer, USA) and the dilution rate was set at 0.03 h^-1 ^for all chemostat experiments. Effluent was removed discontinuously by means of a special overflow device, which has been described previously [82]. The time interval between effluent removals was fixed in such a way that each time approximately 1 % of the culture volume was removed. To prevent excessive foaming, silicone antifoam (10 % vol/vol, BDH Chemicals Ltd, Poole, UK) was discontinuously added at timed intervals. The offgas was cooled by a condensor at 4°C after drying with a Perma Pure dryer (type MD-110-48P-4, Perma Pure, Toms River, USA) oxygen and carbon dioxide concentrations were determined with a NGA 2000 analyser (Rosemount Analytical, Orville, USA). Off-gas flow rates were determined from an average of 10 measurements using a SAGA digital flow meter (Ion Science, Cambridge, UK). Specific rates of carbon dioxide and oxygen consumption were calculated as described previously [83].

### Determination of culture dry weight

Culture samples (10 mL) were filtered over preweighed glass fiber filters (Type A/E, Pall Life Sciences, East Hills, USA). The filters were washed with demineralised water and dried for 20 min at 600 W in a microwave oven and were subsequently weighed.

### Substrate and metabolite analysis

Glucose concentrations in the medium were determined by HPLC using an Aminex HPX-87H column (Biorad, Hercules, USA) at 60°C with 5 mM H_2_SO_4 _as the mobile phase. Phenylacetic acid and penicillinG concentrations were determined by isocratic HPLC using a Platinum EPS C18 column (Alltech, Deerfield, USA) at 30°C. The mobile phase consisted of 5 M acetonitrile with 5 mM KH_2_PO_4 _and 6 mM H_3_PO_4_.

### Sampling and RNA extraction procedures

60 mL of culture broth was sampled and rapidly filtered over a glass fiber filter (Type A/E, Pall Life Sciences, East Hills, USA). The filter with mycelium was wrapped in aluminium foil, quenched in liquid nitrogen and stored at -80°C until further use. For total RNA extraction, half of the pellet was grounded by mortar and pestle under constant cooling with liquid nitrogen. The powder was dissolved in 5 mL of Trizol reagent (Invitrogen) and 1 mL chloroform (Sigma) and mixed well. The two phases were separated by centrifugation (4600 g, 15 min). Total RNA was isolated using a phenol-chloroform extraction method, which consisted of two extraction steps in acid-phenol/chloroform/isoamyl alcohol (5:1, pH 4.8, Ambion, Foster City, USA), followed by a chloroform extraction step. Each time the phases were separated by centrifugation (4600 g, 15 min). Total RNA was precipitated for 30 min at -20°C in 96% ethanol and 0.3 M sodium acetate. The RNA was collected by centrifugation at 23000 g for 15 min and dissolved in RNAse free H_2_O.

### Microarray analysis: probe preparation and target hybridisation

Double stranded cDNA synthesis was carried out using 10 μg of total RNA and the components of the One Cycle cDNA Synthesis Kit (Affymetrix, Santa Clara, USA). The double-stranded cDNA was purified with the GeneChip Sample Cleanup Module (Affymetrix/Qiagen) followed by in vitro transcription and labelling using the GeneChip IVT labeling Kit (Affymetrix). Finally, labelled cRNA was purified (GeneChip Sample Cleanup Module, Affymetrix/Qiagen) prior to fragmentation. 15 μg of fragmented, biotinylated cRNA was hybridised to Affymetrix custom-made *Penicillium chrysogenum *GeneChip^® ^microarrays (array code DSM_PENa520255F) at 45°C for 16 h as described in the Affymetrix users' manual. Washing and staining of arrays were performed using the GeneChip^® ^Fluidics Station 400 and scanning with the Affymetrix GeneArray Scanner 3000.

### Data analysis

Acquisition and quantification of array images were performed using Affymetrix GeneChip Operating Software (GCOS version 1.2). Before comparison, all arrays were globally scaled to a target value of 100 using the average signal from all gene features. To the 15,531 transcript features on the arrays, a filter was applied to extract 13,746 open reading frames of which there were 13,485 different genes. This discrepancy was due to several genes being represented more than once. To represent the variation in triplicate measurements, the coefficient of variation (S.D. divided by the mean) was calculated. When the genes were ranked according to increasing average intensity, the average coefficient of variation showed a sharp increase for the genes with the lowest expression. Therefore, all genes in which the average expression in all conditions was below 12 were removed from the dataset. Subsequently all remaining values below 12 were set to a value of 12. To assess differential expression, the Significance Analysis of Microarrays (SAM version 1.21) add-in to Microsoft™ Excel was used for comparisons of replicate array experiments [37,84]. SAM identifies genes with statistically significant changes in expression by assimilating a set of gene-specific *t *tests. Each gene is assigned a score on the basis of its change in gene expression relative to the standard deviation of repeated measurements for that gene. Genes with scores greater than a threshold are deemed potentially significant. The percentage of such genes identified by chance is the false discovery rate (FDR). To estimate the FDR, nonsense genes are identified by analyzing permutations of the measurements. The threshold can be adjusted to identify smaller or larger sets of genes, and FDRs are calculated for each set [84]. Here internal SAM parameters for fold-change threshold and the false discovery rate values were set at 2 and 1% respectively.

The genes with significantly changed expression in one of the comparisons were arranged in groups via overlapping them in Microsoft™ Excel.

Enrichment of MIPS categories (version 1.3) was assessed by Fisher's Exact test employing hypergeometric distribution with a p-value cut-off of 10^-4 ^(with a Bonferroni correction). The probability was calculated as follows: the p-value of observing z genes, belonging to the same functional category is:

$P=\sum_{x=z}^{\max(N,M)}\frac{\left(\begin{array}{c} N\\ x \end{array}\right)\cdot \left(\begin{array}{c} G-N\\ M-x \end{array}\right)}{\left(\begin{array}{c} G\\ M \end{array}\right)}$, where N is the total number of genes in a category, M is the total number of differentially expressed genes in the cluster and G is the total number of *P. chrysogenum *genes.

Promoter analysis was performed using the web-based software Multiple Em for Motif Elucidation (MEME) [85] incorporated in the software package Genedata Phylosopher (Genedata, Basel, Switzerland). The promoters (from -800, 0) of each set of co-regulated genes were analysed for overrepresented decanucleotides. Promoters with an E-value < 10^-5 ^and without long stretches of A and T (> 40% GC content) were included in the analyses. Consensus sequences were depicted using the web based application WebLogo, version 2.8.2 [86,87]. The transcriptome data analysed in this study have deposited at the Genome Expression Omnibus database  under the accession number GSE12632.

## Authors' contributions

DMH carried out fermentations. DNA microarray analysis and drafted the manuscript, ZAvdK carried out fermentations, PK carried out biochemical analysis, LMR and SH carried out strain construction, MAvdB and RALB coordinated strain construction and biochemical analysis, JTP designed the experiments and helped writing the manuscript. JMD designed the experiments, performed the data analysis, drafted the manuscript and coordinated the study. All authors read and approved the final manuscript.

## Supplementary Material

Additional file 1 **Composition of the Group clusters (Group 1 to 12) as edited in figure 3A.** Each sheet contains the expression data of the genes belonging to the group in the strains DS17690 and DS5066 grown in absence and presence of phenylacetate. The data shown correspond to the average of the triplicate measurements. Click here for file

## References

[B1] Fleming A (1929). On the antibacterial action of cultures of a *Penicillium*, with special reference to their use in the isolation of *B. influenza*. Exp Pathol.

[B2] Lein J, Vanek Z, Hostálek Z (1986). The Panlabs penicillin strain improvement programme. Overproduction of microbial metabolites.

[B3] Peñalva MA, Rowlands RT, Turner G (1998). The optimization of penicillin biosynthesis in fungi. Trends Biotechnol.

[B4] Backus MP, Stauffer JF (1955). The production and selection of a family of strains in *Penicillium chrysogenum*. Mycologia.

[B5] Thykaer J, Nielsen J (2003). Metabolic engineering of beta-lactam production. Metab Eng.

[B6] Barredo JL, van Solingen P, Díez B, Alvarez E, Cantoral JM, Kattevilder A, Smaal EB, Groenen MA, Veenstra AE, Martín JF (1989). Cloning and characterization of the acyl-coenzyme A: 6-aminopenicillanic-acid-acyltransferase gene of *Penicillium chrysogenum*. Gene.

[B7] Barredo JL, Díez B, Alvarez E, Martín JF (1989). Large amplification of a 35-kb DNA fragment carrying two penicillin biosynthetic genes in high penicillin producing strains of *Penicillium chrysogenum*. Curr Genet.

[B8] Zhang JY, Demain AL (1992). ACV Synthetase. Crit Rev Biotechnol.

[B9] Carr LG, Skatrud PL, Scheetz ME, Queener SW, Ingolia TD (1986). Cloning and expression of the isopenicillin N synthetase gene from *Penicillium chrysogenum*. Gene.

[B10] Díez B, Barredo JL, Alvarez E, Cantoral JM, van Solingen P, Groenen MA, Veenstra AE, Martín JF (1989). Two genes involved in penicillin biosynthesis are linked in a 5.1 kb SalI fragment in the genome of *Penicillium chrysogenum*. Mol Gen Genet.

[B11] Díez B, Gutiérrez S, Barredo JL, van Solingen P, Voort LH van der, Martín JF (1990). The cluster of penicillin biosynthetic genes. Identification and characterization of the *pcbAB *gene encoding the alpha-aminoadipyl-cysteinyl-valine synthetase and linkage to the *pcbC *and *penDE *genes. J Biol Chem.

[B12] Gutiérrez S, Fierro F, Casqueiro J, Martín JF (1999). Gene organization and plasticity of the beta-lactam genes in different filamentous fungi. Antonie Van Leeuwenhoek.

[B13] Smith DJ, Burnham MK, Bull JH, Hodgson JE, Ward JM, Browne P, Brown J, Barton B, Earl AJ, Turner G (1990). Beta-lactam antibiotic biosynthetic genes have been conserved in clusters in prokaryotes and eukaryotes. EMBO J.

[B14] Fierro F, Barredo JL, Díez B, Gutiérrez S, Fernández FJ, Martín JF (1995). The penicillin gene cluster is amplified in tandem repeats linked by conserved hexanucleotide sequences. Proc Natl Acad Sci USA.

[B15] Newbert RW, Barton B, Greaves P, Harper J, Turner G (1997). Analysis of a commercially improved *Penicillium chrysogenum *strain series: involvement of recombinogenic regions in amplification and deletion of the penicillin biosynthesis gene cluster. J Ind Microbiol Biotechnol.

[B16] Kamp M van de, Driessen AJ, Konings WN (1999). Compartmentalization and transport in beta-lactam antibiotic biosynthesis by filamentous fungi. Antonie Van Leeuwenhoek.

[B17] Berg MA van den, Albang R, Albermann K, Badger JH, Daran JM, Driessen AJM, Estrada CG, Fedorova ND, Harris D, Heijne W, Joardar V, Kiel J, Kovalchuk A, Martin JF, Niermann WC, Nijland JG, Pronk JT, Roubos JA, Klie I van der, van Peij NNME, Veenhuis M, von Dohren H, Wagner C, Wortman J, Bovenberg RAL (2008). Genome sequencing and analysis of the filamentous fungus *Penicillium chrysogenum*. Nat Biotechnol.

[B18] Gordon M, Pan SC, Virgona A, Numerof P (1953). Biosynthesis of penicillin. I. Role of phenylacetic acid. Science.

[B19] Henriksen CM, Nielsen J, Villadsen J (1998). Modelling of the protonophoric uncoupling by phenoxyacetic acid of the plasma membrane potential of *Penicillium chrysogenum*. Biotechnol Bioeng.

[B20] Hillenga DJ, Versantvoort HJM, Molen S van der, Driessen AJM, Konings WN (1995). *Penicillium chrysogenum *takes up the Penicillin G precursor phenylacetic acid by passive diffusion. Appl Environ Microbiol.

[B21] Arias-Barrau E, Olivera ER, Luengo JM, Fernández C, Galán B, García JL, Díaz E, Miñambres B (2004). The homogentisate pathway: a central catabolic pathway involved in the degradation of L-phenylalanine, L-tyrosine, and 3-hydroxyphenylacetate in *Pseudomonas putida*. J Bacteriol.

[B22] Fernandez-Canon JM, Penalva MA (1995). Molecular characterization of a gene encoding a homogentisate dioxygenase from Aspergillus nidulans and identification of its human and plant homologues. J Biol Chem.

[B23] Ferrer-Sevillano F, Fernández-Cañón JM (2007). Novel *phacB*-encoded cytochrome P450 monooxygenase from *Aspergillus nidulans *with 3-hydroxyphenylacetate 6-hydroxylase and 3,4-dihydroxyphenylacetate 6-hydroxylase activities. Eukayot Cell.

[B24] Mingot JM, Peñalva MA, Fernández-Cañón JM (1999). Disruption of phacA, an *Aspergillus nidulans *gene encoding a novel cytochrome P450 monooxygenase catalyzing phenylacetate 2-hydroxylation, results in penicillin overproduction. J Biol Chem.

[B25] Rodríguez-Sáiz M, Barredo JL, Moreno MA, Fernández-Cañón JM, Peñalva MA, Díez B (2001). Reduced function of a phenylacetate-oxidizing cytochrome P450 caused strong genetic improvement in early phylogeny of penicillin-producing strains. J Bacteriol.

[B26] Rodríguez-Sáiz M, Díez B, Barredo JL (2005). Why did the Fleming strain fail in penicillin industry?. Fungal Genet Biol.

[B27] Eriksen SH, Soderblom TB, Jensen B, Olsen J (1998). Uptake of phenylacetic acid by two strains of *Penicillium chrysogenum*. Biotechnol Bioeng.

[B28] Fernández-Cañón JM, Reglero A, Martínez-Blanco H, Luengo JM (1989). Uptake of phenylacetic acid by *Penicillium chrysogenum *Wis54-1255: a critical regulatory point in benzylpenicillin biosynthesis. J Antibiot (Tokyo).

[B29] Kleijn RJ, Liu F, van Winden WA, van Gulik WM, Ras C, Heijnen JJ (2007). Cytosolic NADPH metabolism in penicillin-G producing and non-producing chemostat cultures of *Penicillium chrysogenum*. Metab Eng.

[B30] van Gulik WM, de Laat WT, Vinke JL, Heijnen JJ (2000). Application of metabolic flux analysis for the identification of metabolic bottlenecks in the biosynthesis of penicillin-G. Biotechnol Bioeng.

[B31] Cantoral JM, Gutiérrez S, Fierro F, Gil-Espinosa S, van Liempt H, Martín JF (1993). Biochemical characterization and molecular genetics of nine mutants of *Penicillium chrysogenum *impaired in penicillin biosynthesis. J Biol Chem.

[B32] Fierro F, Montenegro E, Gutiérrez S, Martín JF (1996). Mutants blocked in penicillin biosynthesis show a deletion of the entire penicillin gene cluster at a specific site within a conserved hexanucleotide sequence. Appl Microbiol Biotechnol.

[B33] Frederiksen RB, Emborg C (1984). Conversion of Cephalosporin-C into 7-phenoxy-acetamido-cephalosporanic acid by acyltransferase of mutants of *Penicillium chrysogenum*. Biotechnol Lett.

[B34] Berg MA van den, Westerlaken I, Leeflang C, Kerkman R, Bovenberg RA (2007). Functional characterization of the penicillin biosynthetic gene cluster of *Penicillium chrysogenum *Wisconsin54-1255. Fungal Genet Biol.

[B35] Harris DM, Diderich JA, Krogt ZA van der, Luttik MA, van Gulik WM, van Dijken JP, Pronk JT (2006). Enzymic analysis of NADPH metabolism in *Penicillium chrysogenum *: presence of a mitochondrial NADPH dehydrogenase in beta-lactam-producing cultures. Metab Eng.

[B36] van Gulik WM, Antoniewicz MR, de Laat WT, Vinke JL, Heijnen JJ (2001). Energetics of growth and penicillin production in a high-producing strain of *Penicillium chrysogenum*. Biotechnol Bioeng.

[B37] Piper MD, Daran-Lapujade P, Bro C, Regenberg B, Knudsen S, Nielsen J, Pronk JT (2002). Reproducibility of oligonucleotide microarray transcriptome analyses. An interlaboratory comparison using chemostat cultures of *Saccharomyces cerevisiae*. J Biol Chem.

[B38] Díez B, Marcos AT, Rodríguez M, de la Fuente JL, Barredo JL (2001). Structural and phylogenetic analysis of the gamma-actin encoding gene from the penicillin-producing fungus *Penicillium chrysogenum*. Curr Microbiol.

[B39] Ruepp A, Zollner A, Maier D, Albermann K, Hani J, Mokrejs M, Tetko I, Guldener U, Mannhaupt G, Munsterkotter M, Mewes HW (2004). The FunCat, a functional annotation scheme for systematic classification of proteins from whole genomes. Nucl Acids Res.

[B40] Fierro F, García-Estrada C, Castillo NI, Rodríguez R, Velasco-Conde T, Martín JF (2006). Transcriptional and bioinformatic analysis of the 56.8 kb DNA region amplified in tandem repeats containing the penicillin gene cluster in *Penicillium chrysogenum*. Fungal Genet Biol.

[B41] Keller NP, Turner G, Bennett JW (2005). Fungal secondary metabolism – from biochemistry to genomics. Nat Rev Microbiol.

[B42] Proctor RH, Hohn TM (1993). Aristolochene Synthase – Isolation, characterization, and bacterial expression of a sesquiterpenoid biosynthetic gene (Ari1) from *Penicillium roqueforti*. J Biol Chem.

[B43] Balunas MJ, Jones WP, Chin YW, Mi QW, Farnsworth NR, Soejarto DD, Cordell GA, Swanson SM, Pezzuto JM, Chai HB, Kinghorn AD (2006). Relationships between inhibitory activity against a cancer cell line panel, profiles of plants collected, and compound classes isolated in an anticancer drug discovery project. Chem Biodivers.

[B44] Martin VJJ, Yoshikuni Y, Keasling JD (2001). The in vivo synthesis of plant sesquiterpenes by *Escherichia coli*. Biotechnol Bioeng.

[B45] Sørensen D, Raditsis A, Trimble LA, Blackwell BA, Sumarah MW, Miller JD (2007). Isolation and structure elucidation by LC-MS-SPE/NMR: PR toxin – and cuspidatol-related eremophilane sesquiterpenes from *Penicillium roqueforti*. J Nat Prod.

[B46] Lamas-Maceiras M, Vaca I, Rodríguez E, Casqueiro J, Martín JF (2006). Amplification and disruption of the phenylacetyl-CoA ligase gene of *Penicillium chrysogenum *encoding an aryl-capping enzyme that supplies phenylacetic acid to the isopenicillin N-acyltransferase. Biochem J.

[B47] Galagan JE, Calvo SE, Cuomo C, Ma LJ, Wortman JR, Batzoglou S, Lee SI, Basturkmen M, Spevak CC, Clutterbuck J, Kapitonov V, Jurka J, Scazzocchio C, Farman M, Butler J, Purcell S, Harris S, Braus GH, Draht O, Busch S, D'Enfert C, Bouchier C, Goldman GH, Bell-Pedersen D, Griffiths-Jones S, Doonan JH, Yu J, Vienken K, Pain A, Freitag M (2005). Sequencing of *Aspergillus nidulans *and comparative analysis with *A. fumigatus *and *A. oryzae*. Nature.

[B48] Snoep JL, Yomano LP, Westerhoff HV, Ingram LO (1995). Protein burden in *Zymomonas mobilis *– Negative flux and growth-control due to overproduction of glycolytic enzymes. Microbiology-Sgm.

[B49] Hazelwood LA, Tai SL, Boer VM, de Winde JH, Pronk JT, Daran JM (2006). A new physiological role for Pdr12p in Saccharomyces cerevisiae: export of aromatic and branched-chain organic acids produced in amino acid catabolism. FEMS Yeast Res.

[B50] Andrade AC, Del Sorbo G, Van Nistelrooy JG, Waard MA (2000). The ABC transporter AtrB from *Aspergillus nidulans *mediates resistance to all major classes of fungicides and some natural toxic compounds. Microbiology.

[B51] Andrade AC, van Nistelrooy JGM, Peery RB, Skatrud PL, De Waard MA (2000). The role of ABC transporters from *Aspergillus nidulans *in protection against cytotoxic agents and in antibiotic production. Mol Gen Genet.

[B52] van Roermund CW, Drissen R, Berg M van den, Ijlst L, Hettema EH, Tabak HF, Waterham HR, Wanders RJ (2001). Identification of a peroxisomal ATP carrier required for medium-chain fatty acid beta-oxidation and normal peroxisome proliferation in *Saccharomyces cerevisiae*. Mol Cell Biol.

[B53] Eichhorn E, Ploeg Jr van der, Kertesz MA, Leisinger T (1997). Characterization of alpha-ketoglutarate-dependent taurine dioxygenase from *Escherichia coli*. J Biol Chem.

[B54] Boer VM, de Winde JH, Pronk JT, Piper MD (2003). The genome-wide transcriptional responses of *Saccharomyces cerevisiae *grown on glucose in aerobic chemostat cultures limited for carbon, nitrogen, phosphorus, or sulfur. J Biol Chem.

[B55] Tai SL, Boer VM, Daran-Lapujade P, Walsh MC, de Winde JH, Daran JM, Pronk JT (2005). Two-dimensional transcriptome analysis in chemostat cultures – Combinatorial effects of oxygen availability and macronutrient limitation in *Saccharomyces cerevisiae*. J Biol Chem.

[B56] Kamp M van de, Pizzinini E, Vos A, Lende TR van der, Schuurs TA, Newbert RW, Turner G, Konings WN, Driessen AJM (1999). Sulfate transport in *Penicillium chrysogenum *: Cloning and characterization of the sutA and sutB genes. J Bacteriol.

[B57] Liang R, Fei YJ, Prasad PD, Ramamoorthy S, Han H, Yang-Feng TL, Hediger MA, Ganapathy V, Leibach FH (1995). Human intestinal H+/peptide cotransporter. Cloning, functional expression, and chromosomal localization. J Biol Chem.

[B58] Daran-Lapujade P, Jansen ML, Daran JM, van Gulik W, de Winde JH, Pronk JT (2004). Role of transcriptional regulation in controlling fluxes in central carbon metabolism of *Saccharomyces cerevisiae*. A chemostat culture study. J Biol Chem.

[B59] Ter Linde JJ, Liang H, Davis RW, Steensma HY, van Dijken JP, Pronk JT (1999). Genome-wide transcriptional analysis of aerobic and anaerobic chemostat cultures of *Saccharomyces cerevisiae*. J Bacteriol.

[B60] Rautio JJ, Smit BA, Wiebe M, Penttilä M, Saloheimo M (2006). Transcriptional monitoring of steady state and effects of anaerobic phases in chemostat cultures of the filamentous fungus *Trichoderma reesei*. BMC Genomics.

[B61] Hua Q, Yang C, Oshima T, Mori H, Shimizu K (2004). Analysis of gene expression in *Escherichia coli *in response to changes of growth-limiting nutrient in chemostat cultures. Appl Environ Microbiol.

[B62] Lee LJ, Barrett JA, Poole RK (2005). Genome-wide transcriptional response of chemostat-cultured to zinc. J Bacteriol.

[B63] Daran-Lapujade P, Daran JM, van Maris AJ, de Winde JH, Pronk JT (2008). Chemostat-Based Micro-Array Analysis in Baker's Yeast. Adv Microb Physiol.

[B64] Galagan JE, Calvo SE, Borkovich KA, Selker EU, Read ND, Jaffe D, FitzHugh W, Ma LJ, Smirnov S, Purcell S, Rehman B, Elkins T, Engels R, Wang SG, Nielsen CB, Butler J, Endrizzi M, Qui DY, Ianakiev P, Pedersen DB, Nelson MA, Werner-Washburne M, Selitrennikoff CP, Kinsey JA, Braun EL, Zelter A, Schulte U, Kothe GO, Jedd G, Mewes W (2003). The genome sequence of the filamentous fungus *Neurospora crassa*. Nature.

[B65] Nierman WC, Pain A, Anderson MJ, Wortman JR, Kim HS, Arroyo J, Berriman M, Abe K, Archer DB, Bermejo C, Bennett J, Bowyer P, Chen D, Collins M, Coulsen R, Davies R, Dyer PS, Farman M, Fedorova N, Fedorova N, Feldblyum TV, Fischer R, Fosker N, Fraser A, Garcia JL, Garcia MJ, Goble A, Goldman GH, Gomi K, Griffith-Jones S (2005). Genomic sequence of the pathogenic and allergenic filamentous fungus *Aspergillus fumigatus*. Nature.

[B66] Machida M, Asai K, Sano M, Tanaka T, Kumagai T, Terai G, Kusumoto K, Arima T, Akita O, Kashiwagi Y, Abe K, Gomi K, Horiuchi H, Kitamoto K, Kobayashi T, Takeuchi M, Denning DW, Galagan JE, Nierman WC, Yu J, Archer DB, Bennett JW, Bhatnagar D, Cleveland TE, Fedorova ND, Gotoh O, Horikawa H, Hosoyama A, Ichinomiya M, Igarashi R (2005). Genome sequencing and analysis of *Aspergillus oryzae*. Nature.

[B67] Genome sequence of *Aspergillus niger*. http://genome.jgi-psf.org/Aspni1/Aspni1.home.html.

[B68] Pel HJ, de Winde JH, Archer DB, Dyer PS, Hofmann G, Schaap PJ, Turner G, de Vries RP, Albang R, Albermann K, Andersen MR, Bendtsen JD, Benen JAE, Berg M van den, Breestraat S, Caddick MX, Contreras R, Cornell M, Coutinho PM, Danchin EGJ, Debets AJM, Dekker P, van Dijck PWM, van Dijk A, Dijkhuizen L, Driessen AJM, D'Enfert C, Geysens S, Goosen C, Groot GSP (2007). Genome sequencing and analysis of the versatile cell factory *Aspergillus niger *CBS 513.88. Nat Biotechnol.

[B69] Genome sequence of *Trichoderma reesei*. http://genome.jgi-psf.org/Trire2/Trire2.home.html.

[B70] Pilsyk S, Natorff R, Sienko M, Paszewski A (2007). Sulfate transport in *Aspergillus nidulans *: A novel gene encoding alternative sulfate transporter. Fungal Genet Biol.

[B71] Giaever G, Chu AM, Ni L, Connelly C, Riles L, Veronneau S, Dow S, Lucau-Danila A, Anderson K, Andre B, Arkin AP, Astromoff A, El Bakkoury M, Bangham R, Benito R, Brachat S, Campanaro S, Curtiss M, Davis K, Deutschbauer A, Entian KD, Flaherty P, Foury F, Garfinkel DJ, Gerstein M, Gotte D, Guldener U, Hegemann JH, Hempel S, Herman Z (2002). Functional profiling of the *Saccharomyces cerevisiae genome*. Nature.

[B72] Tai SL, Snoek I, Luttik MAH, Almering MJH, Walsh MC, Pronk JT, Daran JM (2007). Correlation between transcript profiles and fitness of deletion mutants in anaerobic chemostat cultures of *Saccharomyces cerevisiae*. Microbiology-Sgm.

[B73] Winzeler EA, Shoemaker DD, Astromoff A, Liang H, Anderson K, Andre B, Bangham R, Benito R, Boeke JD, Bussey H, Chu AM, Connelly C, Davis K, Dietrich F, Dow SW, El Bakkoury M, Foury F, Friend SH, Gentalen E, Giaever G, Hegemann JH, Jones T, Laub M, Liao H, Liebundguth N, Lockhart DJ, Lucau-Danila A, Lussier M, M'Rabet N, Menard P (1999). Functional characterization of the *S. cerevisiae *genome by gene deletion and parallel analysis. Science.

[B74] Knijnenburg TA, de Winde JH, Daran JM, Daran-Lapujade P, Pronk JT, Reinders MJT, Wessels LFA (2007). Exploiting combinatorial cultivation conditions to infer transcriptional regulation. BMC Genomics.

[B75] Crawford L, Stepan AM, McAda PC, Rambosek JA, Conder MJ, Vinci VA, Reeves CD (1995). Production of cephalosporin intermediates by feeding adipic acid to recombinant *Penicillium chrysogenum *strains expressing ring expansion activity. Bio-Technology.

[B76] Harris DM, Westerlaken I, Schipper D, Krogt ZA van der, Gombert AK, Sutherland J, Raamsdonk LM, Berg MA van den, Bovenberg RAL, Pronk JT, Daran JM (2009). Engineering of Penicillium chrysogenum for fermentative production of a novel carbamoylated cephem antibiotic precursor. Metab Eng.

[B77] Ullán RV, Campoy S, Casqueiro J, Fernández FJ, Martín JF (2007). Deacetylcephalosporin C production in *Penicillium chrysogenum *by expression of the Isopenicillin N epimerization, ring expansion, and acetylation Genes. Chem Biol.

[B78] Cantoral JM, Díez B, Barredo JL, Alvarez E, Martín JF (1987). High-frequency transformation of *Penicillium chrysogenum*. Bio-Technology.

[B79] de Laat WTAM, Preusting JCG, Koekman BP (2002). Fermentative production of valuable compounds on an industrial scale using chemically defined media. (US2002/0039758).

[B80] Berg MA van den, Bovenberg RAL, Raamsdonk LML, Sutherland JD, de Vroom E, Vollinga RCR (2007). Cephem compound. PCT/NL2004/000367(WO 2004/106347).

[B81] Kohsaka M, Demain AL (1976). Conversion of penicillin N to cephalosporin(S) by cell-free-extracts of *Cephalosporium acremonium*. Biochem Biophys Res Comm.

[B82] van Gulik WM, Meijer JJ, ten Hoopen HJG, Luyben KCAM, Libbenga KR (1989). Growth of a *Catharanthus roseus *cell-suspension culture in a modified chemostat under glucose-limiting conditions. Appl Microbiol Biotechnol.

[B83] van Urk H, Mak PR, Scheffers WA, van Dijken JP (1988). Metabolic responses of *Saccharomyces cerevisiae *CBS 8066 and *Candida utilis *CBS 621 upon transition from glucose limitation to glucose excess. Yeast.

[B84] Tusher VG, Tibshirani R, Chu G (2001). Significance analysis of microarrays applied to the ionizing radiation response. Proc Natl Acad Sci USA.

[B85] Bailey TL, Elkan C (1994). Fitting a mixture model by expectation maximization to discover motifs in biopolymers. Proc Int Conf Intell Syst Mol Biol.

[B86] Crooks GE, Hon G, Chandonia JM, Brenner SE (2004). WebLogo: a sequence logo generator. Genome Res.

[B87] Weblogo, version 2.8.2. http://weblogo.berkeley.edu/logo.cgi.

